# Prenatal diagnosis of Fraser syndrome: a matter of life or death?

**DOI:** 10.1186/s13052-015-0195-6

**Published:** 2015-11-09

**Authors:** Giuseppe De Bernardo, Maurizio Giordano, Antonino Di Toro, Desiree Sordino, Daniele De Brasi

**Affiliations:** Department of Emergency, NICU-AORN Santobono-Pausilipon, Napoli, Italy; Pediatrician Systematic, AORN Santobono-Pausilipon, Napoli, Italy

**Keywords:** Prenatal diagnosis, Cryptophthalmos, Airway abnormalities, Tracheotomy

## Abstract

**Background:**

Fraser Syndrome is a rare, autosomal recessive syndrome. It’s characterized primarily by cryptophthalmos, syndactyly and urogenital malformation. Respiratory malformations are frequently present and not taken into account. To better manage childbirth at the time of delivery it is crucial to get prenatal diagnosis early on in the pregnancy.

**Case presentation:**

We are reporting a female infant born by natural birth with 46,XX. She was characterized phenotypically by cryptophthalmos, syndactyly, bilateral microtia and ambiguous genitalia. A prenatal ultrasound didn’t revealed or raised any suspects for the Fraser Syndrome. It only discovered a unilateral kidney agenesis. At birth the infant showed a severe respiratory distress, intubation was attempted but it failed. The baby was transferred to Santobono-Pausilipon III level hospital. A tracheostomy was performed successfully and saved her life. Computerized Tomography revealed left microphthalmos and a malformation like-coloboma into right ocular globe with cysts and a small calcification parietal anterior. Genetic test revealed the typical mutations in the gene FREM2 confirming the diagnosis of Fraser Syndrome. In her fourth month, after birth, the infant was subjected to an operation to reconstruct eyelids with a mucous membrane graft. The left renal function was normal. The baby showed a delay in motor milestones for visual impairment. At the 19^th^ month fallow-up, during a magnetic resonance it was revealed: a normal morphologic brain development, a thin presence in the right optic nerve and the visual cortex were developing.

**Conclusions:**

The prenatal diagnosis of Fraser Syndrome is frequently possible. The prenatal ultrasound can reveal features like polyhydramnios or oligohydramnios, echogenic lungs, renal abnormalities or agenesis and cryptophthalmos that are pathognomonic of the Fraser Syndrome. The health providers must keep in mind that if there are suspects of the Fraser Syndrome during prenatal exams, the infants could have a severe malformation in the respiratory tract.

## Background

Fraser Syndrome (FS) is a rare, autosomal recessive syndrome phenotypically characterized by multiple malformation with a prevalence of 0,43:100,000 at birth. In 1962, Fraser described a case of two brothers with cryptophthalmos, syndactyly, kidney agenesis, stenosis laryngeal, ambiguous genitalia and malformation at nose and ear [1]. About 250 cases have been reported [2]. Newborns die at birth due to laryngeal malformation, kidney abnormalities or both [3]. When these abnormalities aren’t present, life expectancy are almost normal. Fraser Syndrome is genetically heterogeneous. The mutation in FRAS1, FREM1, FREM2 and GRIP1 are the cause of this pathology [4]. These genes encode the proteins of extracellular matrix that are essentials for the adhesion between basement membrane of epidermis and connective tissues of dermic layer during embryological development. The diagnostic criteria are divided into major criteria (cryptophthalmos, syndactyly, ambiguous genitalia and affected sib) and minor criteria (kidney agenesis, umbilical hernia, congenital malformations of the nose, ear and larynx, cleft lip and palate, skeletal defects and mental retardation) [2]. The diagnosis is confirmed by the presence of two major criteria and one minor criteria or one major and at least four minor criteria. Prenatal diagnosis is possible. Prenatal ultrasound can detect signs as kidney agenesis, oligohydramnios or polyhydramnios and echogenic lungs [5, 6]. Many researchers assert that the cryptophthalmos can be revealed during pregnancy with prenatal ultrasound [7, 8]. Prenatal diagnosis is very important because the infants with FS need particular care in the delivery room. If the prenatal diagnosis is detected early on, health care providers could manage better the respiratory distress of these infants. We report a case of a female infant with Fraser Syndrome undiagnosed during pregnancy. Our infant showed a severe respiratory distress at birth, all the attempts of intubation failed so it was performed tracheotomy. The infant survived because she had a sub-stenosis of larynx.

## Case presentation

**Fig. 1:**
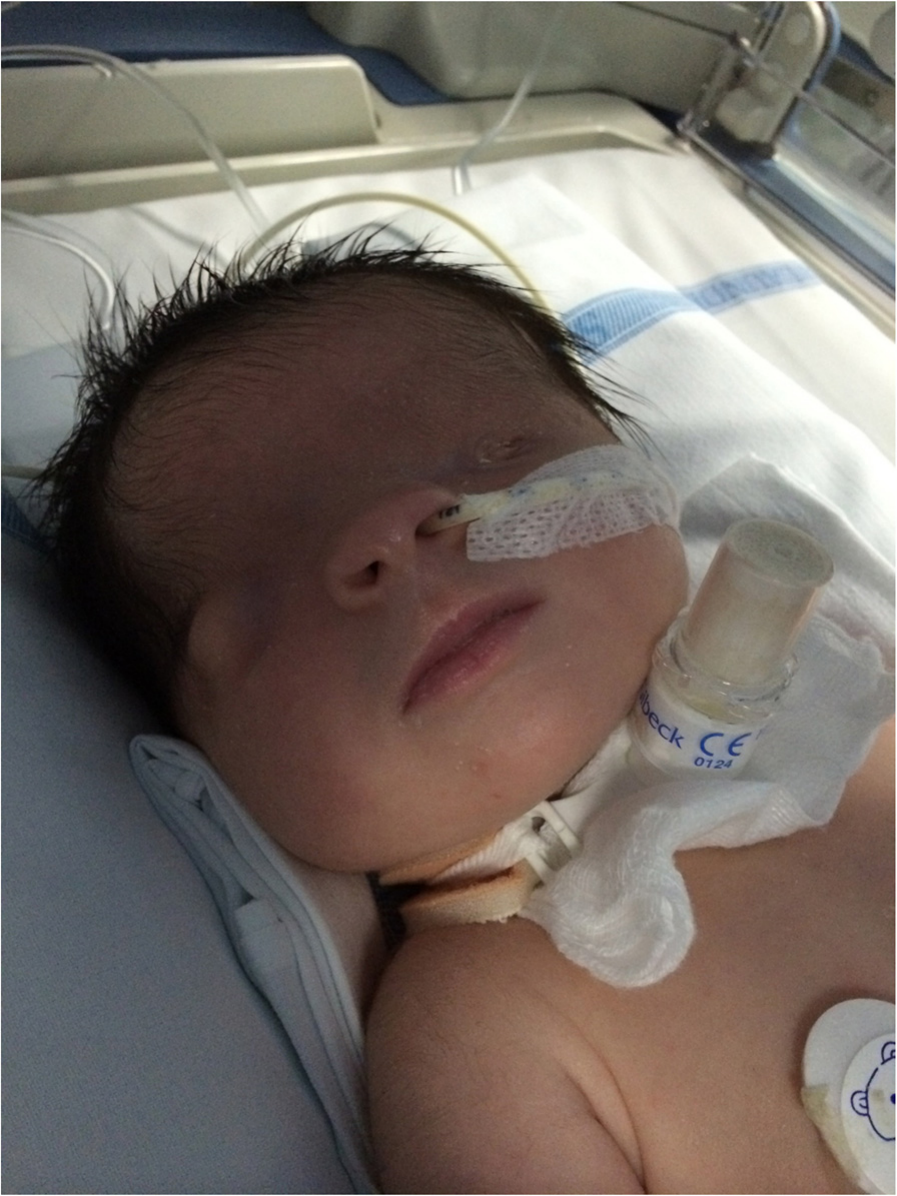
Bilateral cryptophthalmos with microphthalmos into left ocular globe and abnormal right ocular globe

**Fig. 2:**
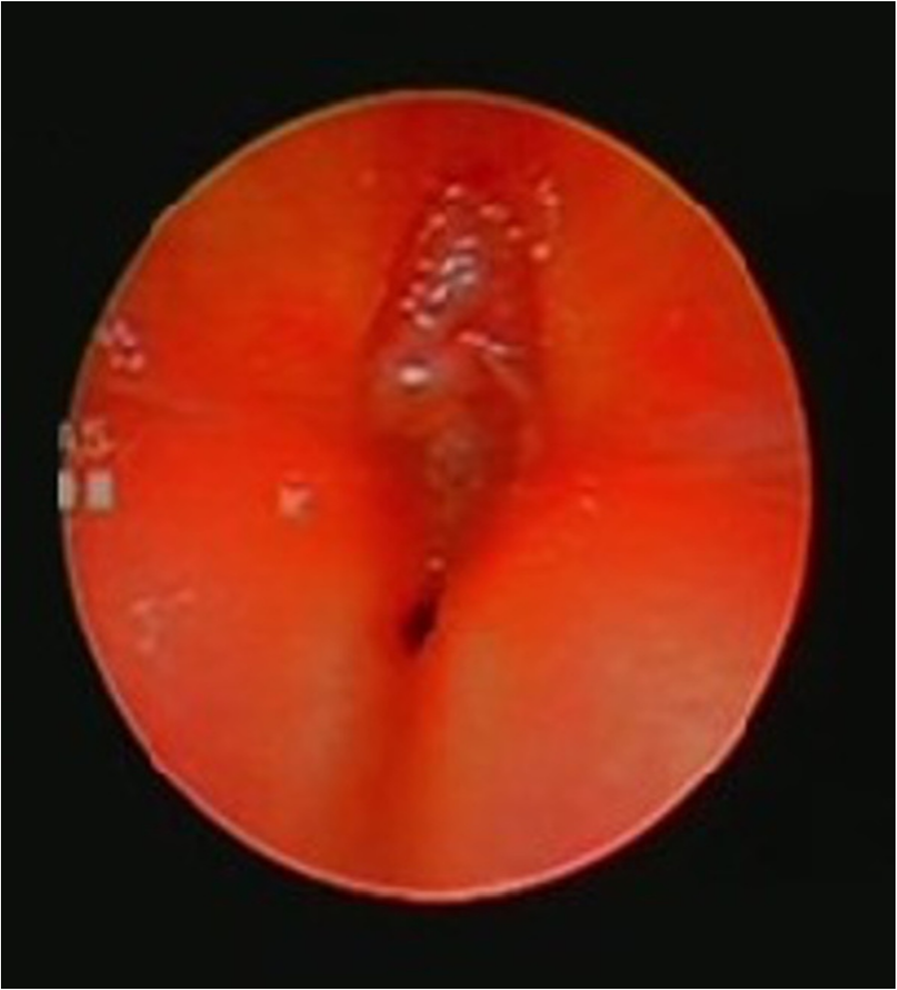
This sub-stenosis of larynx had prevented the intubation

**Fig. 3:**
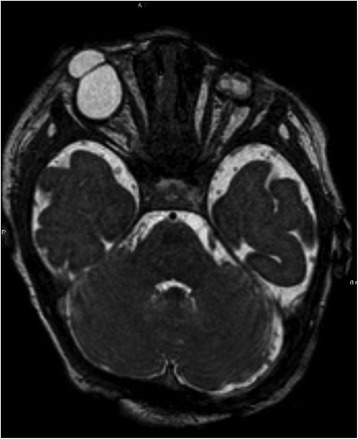
CT revealed microphthalmos into left ocular globe and a structure like-coloboma into right ocular globe

**Fig. 4:**
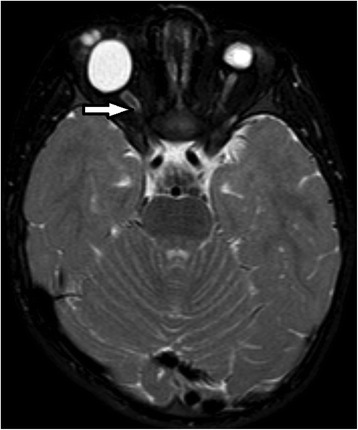
MRI revealed that only the right optic nerve (one *arrow*) was developing

A female infant was born at 40 + 3/7 weeks of gestational age by natural birth after a normal pregnancy. At 22 weeks of gestational age, the ultrasound revealed monolateral kidney agenesis. For this reason, the mother had to undergo for ultrasound controls. A karyotype test was performed and it was normal, 46 XX a diagnosis has not been made. The infant at birth weight was 3220 g, length 46 cm, occipitofrontal circumference 34.5 cm and Apgar score 4/6. Since birth the baby showed cryptophthalmos (Fig. 1), syndactyly, bilateral microtia and ambiguous genitalia. She also showed respiratory distress and bradycardia. The intubation was attempted immediately after birth, but it failed so the baby was transferred to the neonatal intensive care unit at Santobono-Pausilipon hospital. During transport it was performed a nasal intermittent pressure positive ventilation with FiO_2_ 0.40. Oxygen saturation never rose more than 80–82 %. At the neonatal infant care unit fibroscopy intubation was attempted again but it failed due to sub-stenosis laryngeal (Fig. 2) a tracheotomy was performed. The infant started mechanical ventilation with synchronized intermittent positive pressure ventilation because her pH was 7.23 and her pCO_2_ was 68 mmHg until 20^th^ day. FS was suspected. Renal ultrasound confirmed right kidney agenesis and a normal left kidney. The clinical examination confirmed ambiguous genitalia and revealed that the anus was in place, vaginal agenesis, clitoral hypertrophy, ostium in place under the clitoris. Echocardiography revealed mitral valve prolapse and minimal tricuspid regurgitation. Ocular ultrasound showed normal right ocular globe with alterations in the anterior segment and in particular, the crystalline appears small and opaque. It was revealed also high-grade microphthalmos in the left ocular globe with persistent primary vitreous and retinal detachment distal. The Computerized Tomography (CT) revealed left microphthalmos, ocular globe inhomogeneous for structure and signals. The right optic nerve appeared thin. A malformation like-coloboma was present into the right ocular globe with cysts and a small calcification of the parietal anterior (Fig. 3). It was revealed also an incomplete myelination of the brain. Genetic test revealed the mutations c.[5752dup];[8544+1G>T] p.[(Cy51918fs)], in the gene FREM2 confirming the diagnosis of Fraser Syndrome. The infant was operated at 4 month to reconstruct eyelids and after 19 months, mucous membrane grafts were used to line the inner aspect of the flap where there was insufficient conjunctival tissue. It was used a cartilaginous graft to reconstruct lower lid [9]. She showed a delay in motor milestones for the visual impairment, learned to walk at 15 months. In addition, the presence of tracheostomy caused a delay in language. Her outcome improved also thanks to physiotherapist and to neuropsychiatrist. The potential electric visual of the right eye had an amplitude of 0 μV at birth. At sixth month evaluation the potential electric visual was 6.0 μV. Value of left eye was not recorded. Unfortunately, the left eye will probably be replaced by prosthesis. The left renal function was normal although the baby has had a urinary tract infection. A magnetic resonance image (MRI) was performed at 19 months (Fig. 4). It revealed that the right eye had a smaller size than the normal range while left eye dimension was significantly reduced. The right optic nerve and visual cortex were developing. The clinical and instrumental exams didn’t reveal a mental delay.

## Conclusions

FS is a rare disease characterized primarily by cryptophthalmos, syndactyly and urogenital malformation. A different diagnosis may be made with Spears Syndrome, Norrie Syndrome, Meckel Syndrome, Fryns Syndrome and Charge Syndrome. In these syndromes you can find anophthalmia/microphthalmia, genitourinary and lung abnormalities. The malformations as hypoplasia or stenosis of larynx and trachea have been found in 58 % of patients, suggesting that these signs should be promoted among the major signs [2]. The malformations of larynx and trachea can induce the death for severe respiratory distress after birth. Nurullah Okumus have described a case of an infant that is death after birth for anoxia [6]. They knew that prenatal ultrasound revealed oligohydramnios and renal abnormalities but by itself these signs were not enough to diagnosis the FS. In the delivery room intubation and resuscitation failed because the infant had an atresia of larynx so he died due to unidentified FS. To better manage birth in delivery room is crucial prenatal diagnosis. The prenatal ultrasound revealed features like polyhydramnios or oligohydramnios, echogenic lungs, renal abnormalities or agenesis that are pathognomonic of FS [5, 6]. Another important sign was cryptophthalmos it can be revealed during pregnancy. A case of FS in which was observed the cryptophthalmos in high-resolution ultrasound examination was described in 2005 [8]. The ultrasound revealed that the skin was continuous over the eyeballs on both sides and the palpebral fissure could not be identified. In 2012 it was reported the first case in the literature of prenatal diagnosis with bilateral anophthalmia using 3D “reverse face” view ultrasound and magnetic resonance imaging [7]. In our case only a right kidney agenesis was observed during pregnancy but FS was not suspected. Luckily the tracheotomy was performed on time. Health care providers should be always ready to face a potential intubation during childbirth especially in a neonate with FS. For this reason if there is a suspect of FS during pregnancy, the health care providers must keep in mind that the infant could have a severe malformation in respiratory tract [2, 3, 6].

## Consent

“Written informed consent was obtained from the patient for publication of this Case report and any accompanying images. A copy of the written consent is available for review by the Editor-in-Chief of this journal.”

## Abbreviations

FS: Fraser syndrome
CT: computerized tomography
MRI: magnetic risonance image
